# The Influence of Dancesport on College Students’ Rhythm Perception Ability

**DOI:** 10.3390/brainsci16020238

**Published:** 2026-02-19

**Authors:** Qinran Yu, Xinhong Jin, Yingying Wang

**Affiliations:** 1 Faculty of Health Sciences and Sports, Macao Polytechnic University, Macao 999078, China; p2417165@mpu.edu.mo; 2 School of Psychology, Shanghai University of Sport, Shanghai 200438, China

**Keywords:** college students, dancesport, exercise intervention, rhythm perception

## Abstract

**Objective:** This study investigated the effects of a 10-week dancesport intervention on rhythm perception ability and its multisensory correlates in college students. **Methods:** Forty students were randomly assigned to an intervention group (dancesport) and a control group (Health Qigong). Rhythm perception was assessed across different task difficulties (Experiment 1) and through somatosensory, visual, and auditory channels (Experiment 2). Data were analyzed using repeated-measures ANOVA with Bonferroni-corrected pairwise comparisons. **Results:** The dancesport group showed significant improvement in rhythm perception accuracy at all difficulty levels. Furthermore, they achieved superior post-test performance compared to the control group on pooled-difficulty tasks. Significant enhancements were also found in somatosensory reaction time and in both the accuracy and reaction time of auditory processing. Visual channel improvement was restricted to accuracy. **Conclusions:** A 10-week dancesport training program led to significant within-group improvement in rhythm perception across all difficulty levels and enabled superior performance relative to the control group on pooled-difficulty tasks. It also enhances multisensory processing, particularly in somatosensory and auditory channels.

## 1. Introduction

Rhythmic perception is a core human capacity for temporal processing, relying on both interval-based timing (the ability to distinguish absolute duration differences) and beat-based timing (the ability to measure regular intervals) [1]. The former involves the direct encoding of duration, while the latter demonstrates predictive grasp of rhythmic structures. Beat-based timing is thought to be a human advantage [2], enabling the dynamic prediction and synchronization of movement with external rhythms, as in dance [3,4]. However, rhythmic perception transcends auditory processing; it involves cross-modal integration of perception, action, and cognition. Theoretically, embodied cognition posits that motor execution and rhythmic representation share sensory–motor circuits, where physical actions serve not only as skill carriers but also as cognitive tools for internalizing temporal rules [5]. Resonance theory further suggests that rhythmic processing involves the alignment of internal timing mechanisms with external rhythms [6]. However, the effect of motor skill training on rhythm perception, especially the promotion of two kinds of timing processing, needs further study.

Rhythm perception relies not only on auditory input but also involves visual and somatosensory modality. While previous studies have primarily focused on auditory rhythm perception, research on visual and somatosensory timing processing remains limited [7]. Existing evidence indicates that motor experience plays a crucial role in the development of rhythm perception. Research demonstrated [8] that physical movement experience significantly influences auditory rhythm encoding in infants, while runners and cyclists primarily regulate somatosensory rhythm through closed-loop feedback during training, highlighting the pivotal role of motor experience in rhythm initiation and adaptive adjustments. However, maintaining rhythmic stability exhibits specific effects influenced by movement types [9]. This suggests that motor experience enhances the integration of actions and multi-channel rhythmic information through a cross-modal prediction-correction mechanism [10]. As a comprehensive sport integrating music, movement, and artistic expression, ballroom dance features diverse rhythmic patterns and high spatiotemporal complexity in movements. During dance, practitioners must simultaneously process multiple perceptual stimuli, including musical beats, dynamic force variations, and partner coordination, involving auditory, visual, and somatosensory modality in rhythmic perception. The musical rhythm of Cha-cha can reach 27–32 beats per minute, with five movements completed within four beats. One study has shown that dancing requires participants to rapidly parse rhythmic structures and coordinate body movements under dynamic auditory–motor integration demands [11]. A neuroimaging study has observed enhanced functional connectivity between auditory and motor regions in professional dancers, which may support more efficient processing of rhythmic information [12]. Behavioral experiments further revealed that dancers prioritize extracting rhythmic periodicity during movement execution [13], dynamically adjusting movement timing through predictive-correction strategies [14]. This rhythmic anticipation ability enables them to maintain movement stability in complex musical environments while significantly enhancing synchronization in duet performances. A cross-sectional study comparing dancesport athletes with college students demonstrated higher rhythmic synchronization accuracy in athletes [15]. These findings indicate that dancesport not only promotes multisensory rhythmic processing but also creates training advantages via perceptual-motor coupling and predictive-correction mechanisms. This implies that systematic dancesport interventions may transfer this rhythmic perception advantage to non-professional populations.

Although existing studies have demonstrated that professional dancers excel in rhythm perception and movement synchronization, most research has focused on professional athletes or single-channel rhythm tasks, predominantly employing cross-sectional designs with limited longitudinal intervention evidence in non-professional populations. Given that college students are in early adulthood with remaining brain plasticity [16], this provides a physiological foundation for enhancing complex cognitive skills such as rhythm perception.

Therefore, the aim of this study was to investigate the effects of a structured dancesport intervention on rhythm perception in college students. We addressed two specific research questions: First, can dancesport training improve rhythm perception ability? Second, does such training enhance rhythm processing across different sensory channels? To this end, we conducted a longitudinal intervention study with two experiments: Experiment 1 assessed rhythm perception acuity at varying difficulty levels using a time discrimination task, and Experiment 2 evaluated rhythm processing within somatosensory, visual, and auditory channels to examine cross-modal transfer effects.

## 2. Experiment 1: The Impact of Dancesport on Rhythm Perception Ability of College Students

### 2.1. Subjects and Methods

#### 2.1.1. Study Subjects

A total of 40 full-time undergraduate students were recruited and screened, and the participants were divided into an intervention group (dancesport) and a control group (Health Qigong), with 20 participants in each group. Both groups were required to maintain their original exercise habits during the experiment. The sample size was calculated using the G-power 3.1, with a medium effect size (*η*^2^*_p_* = 0.25), an expected power (1 − *β* = 0.80), and a significance level (*α* = 0.05). The required sample size was calculated to be 34 participants (17 in each group), and the actual sample size met the requirements.

Randomization Procedure: Eligible participants (N = 40) were randomly allocated in a 1:1 ratio to either the experimental (dancesport) group or the control (Health Qigong) group using a computer-generated random number sequence. The allocation list was concealed from the recruiters and assessors by a researcher not involved in enrollment or evaluation until after group assignment was completed, ensuring allocation concealment.

The study participants were full-time undergraduate students who had no surgical history, systematic sports training history, or systematic music learning history within the past three months, and were proficient in computer operation. All subjects were familiar with the basic procedures of the experiment, had no prior history of mental disorders or neurological dysfunction, exhibited normal visual or corrected visual acuity, and were free from color vision disorders. They were all right-handed and had no prior experience with similar experiments. The basic demographic information of the participants in each group is shown in Table 1.

Before the intervention, participants’ level of physical activity was assessed using the Chinese version of the International Physical Activity Questionnaire—Short Form (IPAQ-SF), which has demonstrated good reliability and validity in Chinese populations [17]. Participants reported the frequency (days per week) and duration (minutes per day) spent on walking, moderate-intensity, and high-intensity activities in the past 7 days. According to the standard IPAQ scoring protocol, total weekly physical activity volume was calculated as metabolic equivalent (MET) minutes per week (MET-min/week) using the formula: Total MET-min/week = (8.0 × weekly high-intensity activity duration) + (4.0 × weekly moderate-intensity activity duration) + (3.3 × weekly walking duration). All self-reported time data for prior experiences in music, dance, and rhythm games are presented in minutes per week (min/week).

#### 2.1.2. Exercise Intervention Program

The experimental group underwent a 10-week dancesport intervention program designed by a certified professional dancesport coach. Sessions lasted 90 min and were held twice per week. Each session began with a 5-min warm-up involving light aerobic movements and dynamic stretching to music with a steady beat (110 BPM), followed by 75 min of core practice focused on three foundational ballroom dances—Waltz (3/4 time, 28–30 bars/min), Tango (2/4 or 4/4 time, 30–32 bars/min), and Cha-Cha (4/4 time, 30–32 bars/min)—all practiced individually without partner work. Training emphasized real-time synchronization of full-body movements with the musical beat, progressing from basic step patterns to simple variations and turns. Sessions concluded with 5 min of cool-down stretching. The program was designed to be accessible and progressively challenging for participants without prior dance experience. The control group underwent a 10-week Health Qigong intervention matched in duration and frequency (90 min, twice weekly), led by a qualified Health Qigong instructor. Each session included a 5-min warm-up (gentle joint mobilization), 75 min of guided practice in Baduanjin (a standard set of eight gentle, flowing exercises emphasizing slow movement, coordinated breathing, and internal focus with minimal external rhythmic cues), and a 5-min cool-down (quiet standing and breathing). Intervention adherence was monitored throughout the study, and all participants in both the experimental and control groups completed the full program with no sample attrition.

The control group underwent a Health Qigong exercise intervention. The selection of Health Qigong as an active control was deliberate. While both interventions were conducted with background music, they differed fundamentally in their rhythmic demands. Dancesport requires obligatory and precise synchronization of movement to the explicit, often complex meter and tempo of the music. In contrast, Health Qigong movements are self-paced, fluid, and continuous; the accompanying music is slow and atmospheric, and strict alignment with the beat is not required. This design allows us to isolate the specific effects of strict auditory–motor rhythmic synchronization inherent in dancesport, while controlling for general factors such as physical activity duration, group engagement, and the presence of auditory stimulation. Furthermore, as a structured, engaging, and socially conducted group activity, Health Qigong served to match the experimental group on key experiential factors such as perceived task value, social interaction, and overall participant motivation, thereby helping to control for potential confounding differences in engagement and adherence beyond the core rhythmic element.

The 10-week duration was selected based on evidence from previous studies indicating that physical activity training lasting between 8 and 10 weeks is effective for achieving desirable health and cognitive outcomes [18,19].

#### 2.1.3. Experimental Procedures

The study employed a 2 × 2 × 3 factorial design (group: experimental vs. control; time: pretest vs. posttest; task difficulty: Pooled-difficulty, medium-difficulty, high-difficulty tasks). With group as the between-subjects factor and time as the within-subjects factor, the dependent variables were the correct response rates for rhythm perception tasks across three difficulty levels. The experimental protocol comprised three phases: pretest, a 10-week exercise intervention, and posttest.

Due to the overt differences in movement forms and rhythmic demands between the dancesport and Health Qigong interventions, blinding of participants and instructors was not feasible. To minimize measurement bias, a single-blind assessment procedure was employed: all research assistants conducting the pre- and post-test assessments were kept unaware of participants’ group allocation and were not involved in the intervention delivery. Furthermore, the rhythm perception tasks were administered and responses recorded automatically by the E-Prime 3.0 software, which helped ensure objective data collection.

##### Rhythm Perception Measurement

**Figure 1:**
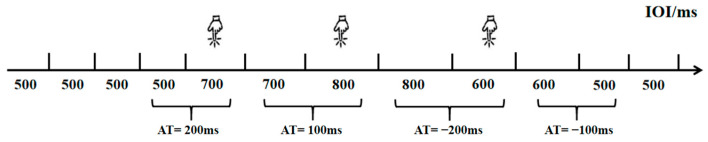
Flowchart of the Rhythm Perception Experiment.

Building upon previous research [20], this experiment employed a rhythm perception task (Task 1) to measure college students’ rhythm processing abilities. This task was designed to specifically assess the perceptual component of rhythm processing—namely, the ability to discriminate subtle changes in temporal intervals within an auditory sequence—while minimizing the influence of motor execution. Participants were not required to produce any rhythmic movements in sync with the stimuli. The task was programmed and data collected using the E-prime 3.0 psychological experiment software. Participants were instructed to determine whether the inter-onset interval (IOI) of consecutive sound stimuli had changed from the previous one, pressing the D key if a change occurred and holding the key if no change was detected. The experimental procedure is detailed in Figure 1. The first four sound stimuli were presented with a 500 ms initial IOI. Starting from the fifth stimulus, the IOI was randomly altered—either maintained at 0 ms with uniform speed or accelerated/decelerated by 100 ms or 200 ms increments. Each rhythm change was followed by at least one uniform IOI to prevent participants from becoming disoriented or delaying responses due to continuous rhythm variations. The task consisted of 71 trials, including 7 practice trials and 64 formal trials, which were divided into two blocks of 32 trials each. Different pitch audio was selected for the two intervals to avoid fatigue effects caused by repeated identical sounds, with at least 30 s of rest between intervals. To test the impact of task difficulty, the IOI variations included two levels: a medium-difficulty task with ΔT = 200 ms and a high-difficulty task with ΔT = 100 ms. In the rhythm perception task, the effective response window is defined as the entire duration from the end of the n-th note to the onset of the next rhythmic change (n > 5), ensuring that at least one uniform note (n + 1) follows the n-th note. This design provides sufficient listening opportunities while preventing delayed responses from being incorrectly counted as the next rhythmic change due to an excessively short response window. The experimental variations include equal halves of increasing and decreasing directions. To ensure precise stimulus presentation and response collection, the task was programmed using E-prime 3.0 software, which provides millisecond-level timing accuracy on standard Windows systems. Participants responded using a standard keyboard in a controlled laboratory environment. This setup allows for the reliable measurement of pure perceptual timing ability, independent of motor speed variability.

The total number of trials was optimized from the original protocol to suit the longitudinal design and non-professional participant pool, aiming to preserve task sensitivity while preventing fatigue.

#### 2.1.4. Data Statistics and Analysis

Statistical analysis was performed using SPSS 26.0 software [21]. The independent samples *t*-test was employed to assess differences between the experimental and control groups in the pretest. A repeated-measures ANOVA was used to evaluate the intervention effects, with a statistical significance threshold of α < 0.05.

For significant interactions, simple effect analyses (pairwise comparisons) were conducted. To control for Type I error inflation, the Bonferroni correction was applied to each set of four pairwise comparisons within a significant interaction. The adjusted significance level was α = 0.0125 (0.05/4).

Effect sizes were reported as follows: for repeated-measures ANOVA, partial eta-squared (*η*^2^*_p_*) was calculated to quantify the magnitude of main effects and interactions, with conventional thresholds of 0.01 (small), 0.06 (medium), and 0.14 (large). For pairwise comparisons derived from simple effect analyses, Cohen‘s *d* was computed, with thresholds of 0.2 (small), 0.5 (medium), and 0.8 (large) [22].

### 2.2. Research Findings and Analysis

#### 2.2.1. Baseline Test Comparison Analysis Between Groups Before Intervention

An independent samples T-test was performed on the pre-test data of rhythmic perception ability across sensory channels in both the experimental and control groups. The results showed no significant differences in the correct rates of undifferentiated, moderate, and high-difficulty rhythmic perception tasks between the two groups (Table 2), indicating consistent baseline levels.

#### 2.2.2. The Influence of Dancesport on Rhythm Perception Ability of College Students

A 2 (group: experimental vs. control) × 2 (time: pretest vs. posttest) × 3 (task difficulty: low, medium, high) mixed-design repeated-measures ANOVA was conducted to analyze the accuracy rates. The results revealed significant interaction effects between task difficulty and time [*F*(2,228) = 3.07, *p* < 0.05, *η*^2^*_p_* = 0.03], as well as between group and time [*F*(1,228) = 24.19, *p* < 0.001, *η*^2^*_p_* = 0.10]. However, the interaction effect of task difficulty × group × time was not statistically significant [*F*(2,228) = 0.26, *p* = 0.768, *η*^2^*_p_* = 0.00]. To further investigate the impact of dancesport intervention on rhythm processing ability under varying difficulty conditions, repeated-measures ANOVA was performed on accuracy rates across different difficulty levels.

A repeated-measures ANOVA was conducted to investigate the effect of dancesport on the accuracy of rhythm perception among college students across the pooled-difficulty levels. The results revealed that the main effect of group was not statistically significant [*F*(1,38) = 0.72, *p* = 0.403, *η*^2^*_p_* = 0.02], while the main effect of time was significant [*F*(1,38) = 33.53, *p* < 0.001, *η*^2^*_p_* = 0.47]. Additionally, the interaction between group and time was statistically significant [*F*(1,38) = 9.17, *p* < 0.001, *η*^2^*_p_* = 0.19].

Simple effect analysis with Bonferroni-adjusted comparisons revealed that the post-test accuracy of the experimental group was significantly higher than that of the pre-test (*p* < 0.001, adjusted, Cohen’s *d* = 1.84); the control group showed no significant difference between pre-test and post-test (*p* = 0.058, adjusted, *d* = 0.64); in the pre-test, the difference in accuracy between the experimental group and the control group was not significant (*p* = 0.14, adjusted, *d* = −0.50); in the post-test, the accuracy of the experimental group was significantly higher than that of the control group (*p* = 0.009, adjusted, *d* = 0.80).

**Figure 2:**
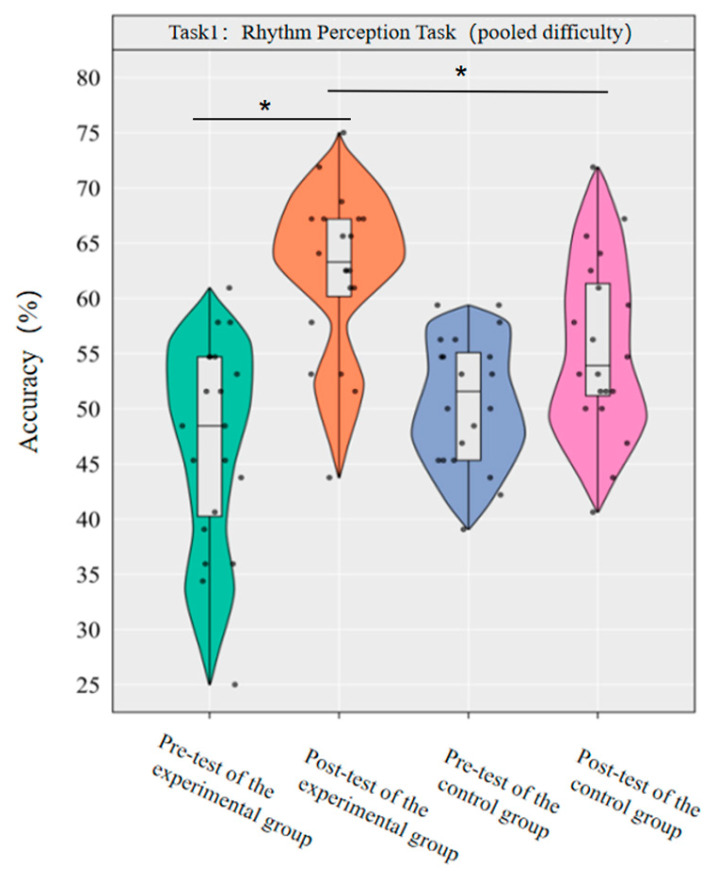
Comparison of the accuracy rates of the two groups of pooled-difficulty rhythm perception before and after tests. Note: * *p* < 0.0125.

This demonstrates that a 10-week dancesport intervention can effectively enhance college students’ rhythm perception ability across the pooled-difficulty levels (Figure 2).

A repeated-measurement ANOVA was conducted to investigate the effect of dancesport on the accuracy of medium-difficulty rhythm perception among college students. The results revealed that the main effect of group was not significant [*F*(1,38) = 0.00, *p* = 0.981, *η*^2^*_p_* = 0.00], while the main effect of time was significant [*F*(1,38) = 43.64, *p* < 0.001, *η*^2^*_p_* = 0.54]. Additionally, the interaction between group and time was statistically significant [*F*(1,3) = 8.75, *p* < 0.01, *η*^2^*_p_* = 0.19].

Simple effect analysis with Bonferroni-adjusted comparisons revealed that the post-test accuracy rate of the experimental group was significantly higher than that of the pre-test (*p* < 0.001, adjusted, Cohen’s *d* = 2.08); the pre-post difference in the control group was not significant (*p* = 0.014, adjusted, *d* = 0.08); in the pre-test, the difference in accuracy rates between the experimental group and the control group was not significant (*p* = 0.068, adjusted, *d* = −0.64); in the post-test, the difference in accuracy rates between the experimental group and the control group was not significant (*p* = 0.059, adjusted, *d* = 0.64).

**Figure 3:**
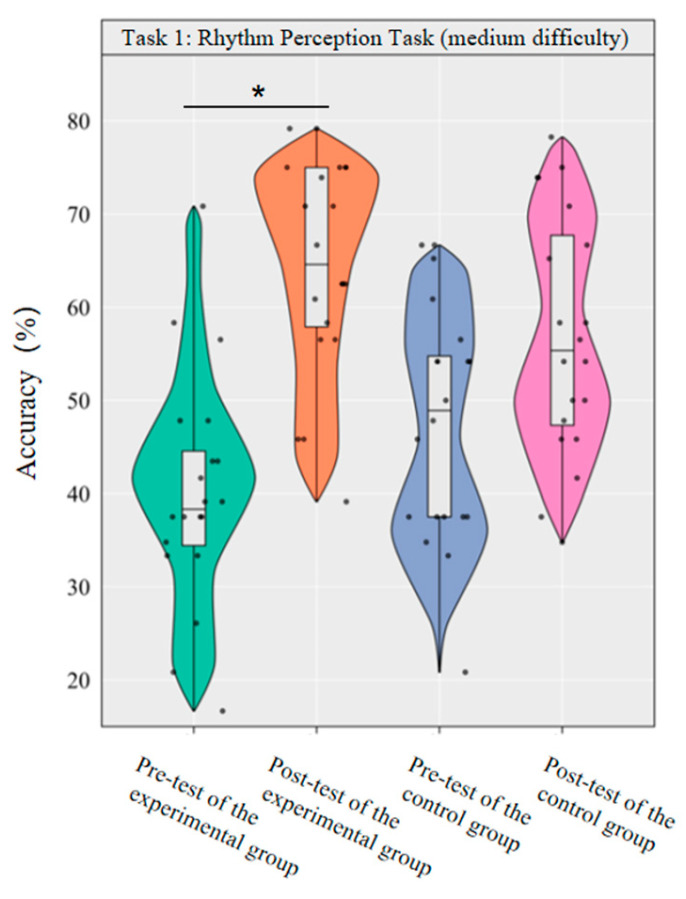
Comparison of the accuracy rates of the two groups of medium-difficulty rhythm perception before and after tests. Note: * *p* < 0.0125.

This demonstrates that a 10-week dancesport intervention can effectively enhance college students’ own rhythmic perception performance in medium-difficulty tasks, as shown by significant within-group improvement (Figure 3).

A repeated-measurement ANOVA was conducted to investigate the effect of dancesport on the accuracy of high-difficulty rhythm perception among college students. The results revealed that the main effect of group was not significant [*F*(1,38) = 0.12, *p* = 0.726, *η*^2^*_p_* = 0.00], while the main effect of time was significant [*F*(1,38) = 12.09, *p* < 0.001, *η*^2^*_p_* = 0.24]. Additionally, the interaction between group and time was statistically significant [*F*(1,38) = 7.38, *p* < 0.05, *η*^2^*_p_* = 0.16].

Simple effect analysis with Bonferroni-adjusted comparisons revealed that the post-test accuracy of the experimental group significantly increased compared to the pre-test (*p* < 0.001, adjusted, Cohen’s *d* = 1.33); the control group showed no significant difference between pre- and post-tests (*p* = 0.594, adjusted, *d* = 0.21). In the pre-test, the difference in accuracy between the experimental and control groups was not significant (*p* = 0.067, adjusted, *d* = −0.52). The post-test group difference was not significant (*p* = 0.023, adjusted, *d* = 0.76).

**Figure 4:**
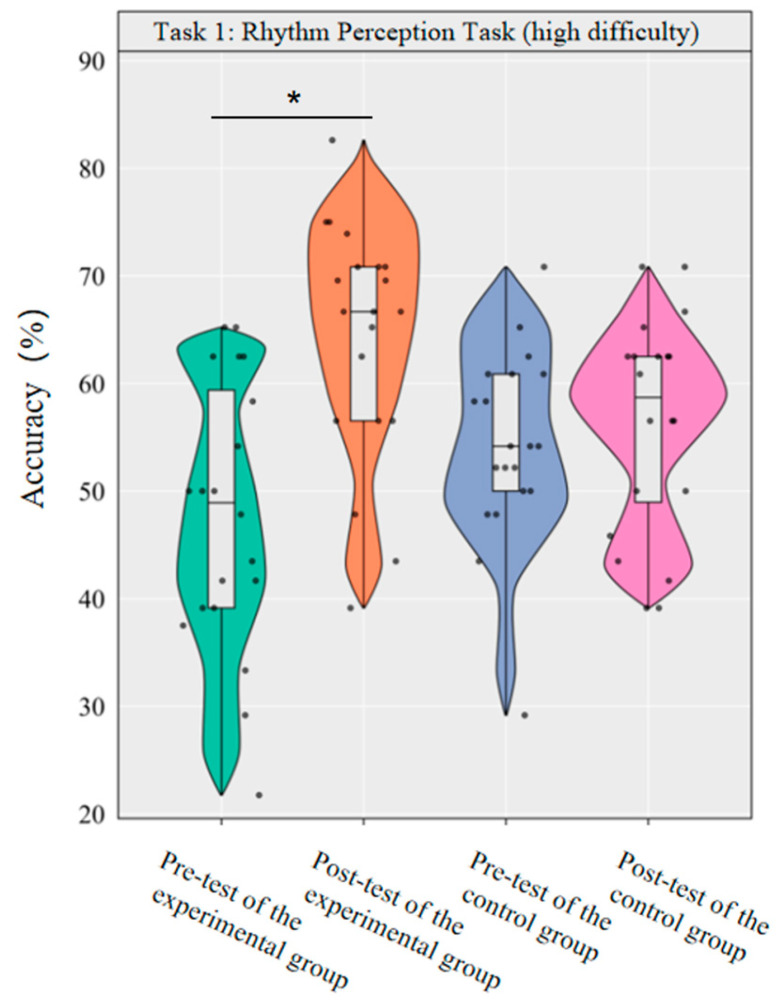
Comparison of the accuracy rates of the two groups of high-difficulty rhythm perception before and after tests. Note: * *p* < 0.0125.

This demonstrates that a 10-week dancesport intervention can effectively enhance college students’ own rhythmic perception performance in high-difficulty tasks, as shown by significant within-group improvement (Figure 4).

The results show that the 10-week dancesport intervention can effectively improve the rhythm perception ability of college students. This improvement was consistent across task difficulty levels, as indicated by significant within-group gains in the experimental group and superior performance relative to the control group in pooled-difficulty tasks.

## 3. Experiment 2: The Impact of Dancesport on Rhythmic Perception Capabilities of Multiple Sensory Channels in College Students

### 3.1. Subjects and Methods

#### 3.1.1. Study Subjects

The same as experiment 1.

#### 3.1.2. Exercise Intervention Program

The same as experiment 1.

#### 3.1.3. Experimental Procedures

A 2 × 2 (group: experimental group vs. control group; time: pretest vs. posttest) mixed design was employed, with group serving as the between-subjects factor and time as the within-subjects factor. The dependent variables included duration of the somatosensory modality rhythm perception task, accuracy and reaction time of the visual channel rhythm perception task, and accuracy and reaction time of the auditory channel rhythm perception task. The experiment comprised three phases: pretest, 10-week exercise intervention, and posttest. Trials exceeding or falling below ±3 standard deviations were excluded.

##### Measurement of Rhythm Perception in the Somatosensory Modality

The rhythm perception of the somatosensory modality was measured through a behavioral experiment (Task 2). In contrast to the pure perception tasks, this task integrates perception with motor execution to evaluate sensorimotor synchronization and multi-limb coordination under rhythmic guidance. Here, the rhythm perceived through the auditory channel must be simultaneously translated into coordinated actions, reflecting a key aspect of dancesport performance. Participants listened to the tango music “He is a Pirate” and started timing from the moment the music began. They were instructed to clap in sync with the rhythm, perform a stationary foot–toe lift, and chant the beat count. The timing stopped after completing four consecutive eight-beat rhythms accurately. A stopwatch was used throughout the process. The evaluation metric was the time it took for participants to correctly identify the rhythm of four eight-beat rhythms while simultaneously completing the four rhythmic actions. Shorter times indicated stronger somatosensory modality rhythm perception.

This task is conceptualized as a somatosensory rhythm perception task with an integrated action component. The rationale is that in the somatosensory modality, rhythm perception is inherently action-oriented; it is demonstrated and measured through the ability to synchronize one’s movements to an external rhythmic cue. The requirement to accurately clap, lift the foot, and vocalize in time necessitates precise internal representation and continuous tracking of the musical beat. Therefore, the time to correctly complete the sequence reflects the efficiency and accuracy of this internal rhythm representation in guiding real-time, multi-limb motor synchronization. While motor execution is involved, it is the rhythmic percept that dictates the timing, making the task a valid measure of functional rhythm perception in the somatosensory–motor domain.

##### Measurement of Rhythm Perception in Visual Channels

**Figure 5:**
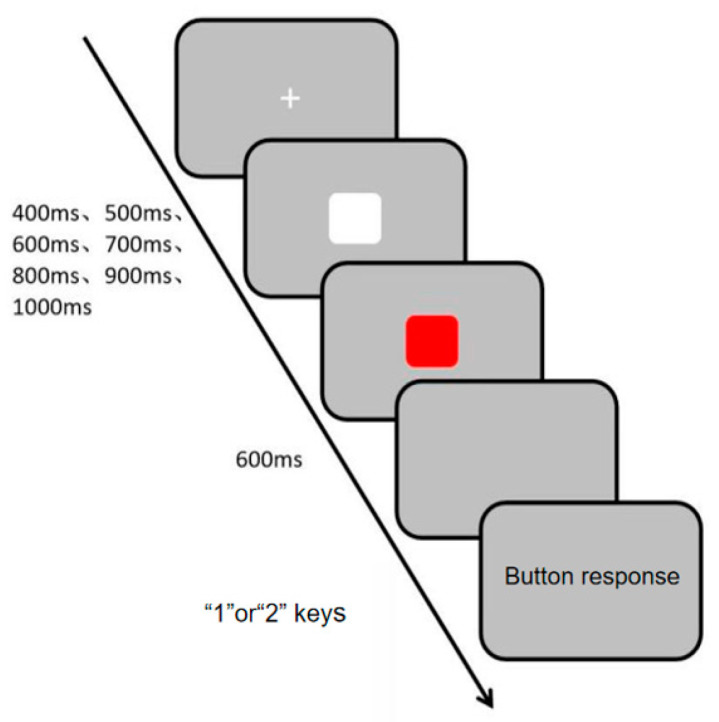
Flowchart of Visual Channel Rhythm Perception Task.

Visual channel rhythm perception was measured using the Visual Channel Rhythm Perception Task (Task 3), programmed and data collected with the E-prime 3.0 psychological experiment software. During the experiment, a white “+” fixation point appeared in the center of the screen, followed by white and red squares presented sequentially at random intervals of 400 ms, 500 ms, 600 ms, 700 ms, 800 ms, 900 ms, and 1000 ms. Participants were informed that the two squares would appear at different times but not the exact intervals. They were instructed to make their judgment after the second square disappeared, ensuring that the response was based on a completed perceptual evaluation rather than a speeded reaction to an ongoing stimulus. This design emphasizes accuracy in temporal discrimination over simple reaction speed. They were instructed to press the “1” or “2” key to determine if the squares appeared at the same time (press “1” for consistency, “2” for inconsistency). The specific experimental procedure is shown in Figure 5. The experiment consisted of 10 practice trials and 30 main trials. Evaluation metrics included accuracy rate and reaction time, with higher accuracy and shorter reaction time indicating stronger visual channel rhythm perception ability.

The task procedure and instructions were reviewed for clarity and appropriateness by two experts in sports psychology prior to the experiment. The trial number for this perceptual discrimination task was set in line with standard practices in the field to ensure a reliable measure of performance while maintaining participant engagement across the multi-experiment session.

##### Measurement of Rhythmic Perception in the Auditory Channel

**Figure 6:**
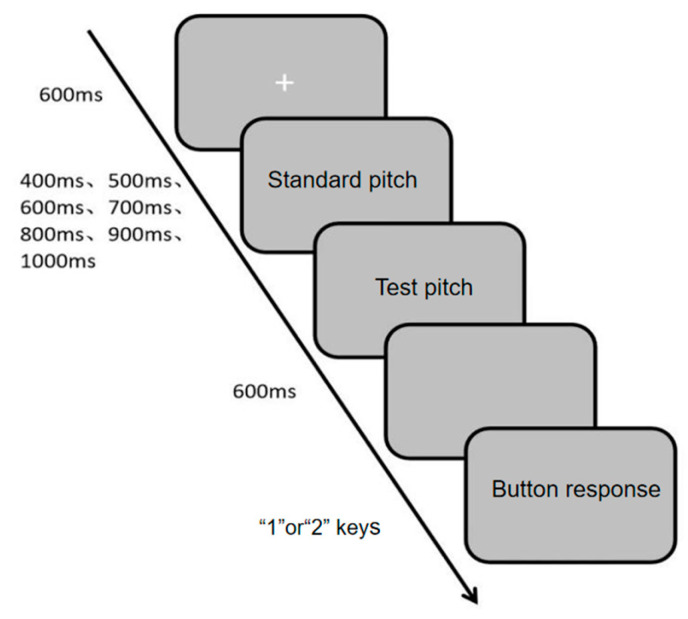
Flowchart of Auditory Channel Rhythm Perception Task.

The auditory channel rhythm perception was measured using the Auditory Channel Rhythm Perception Task (Task 4), programmed and data collected with the E-prime 3.0 psychological experiment software. During the experiment, a white “+” fixation point appeared in the center of the screen, followed by two-tone sequences (standard tone and test tone) played sequentially. The presentation durations of the two tones were randomly selected from 400 ms, 500 ms, 600 ms, 700 ms, 800 ms, 900 ms, and 1000 ms. Participants were informed of the different presentation durations before the experiment but not the specific durations. Similarly, the judgment (key press) was made after the offset of the second tone sequence. This post-stimulus response window ensures that measured reaction time reflects the speed of the perceptual decision process (comparing the memorized durations) rather than the latency of initiating a synchronized movement. They were instructed to press the “1” or “2” key to determine whether the two-tone sequences were presented at the same time. If consistent, they pressed “1”; if inconsistent, they pressed “2”. The detailed experimental procedure is shown in Figure 6. The experiment consisted of 10 practice trials and 30 main trials. The evaluation metrics were accuracy rate and reaction time, with higher accuracy and shorter reaction time indicating stronger auditory channel rhythm perception ability.

The task procedure and instructions were reviewed for clarity and appropriateness by two experts in sports psychology prior to the experiment. The trial number for this perceptual discrimination task was set in line with standard practices in the field to ensure a reliable measure of performance while maintaining participant engagement across the multi-experiment session.

#### 3.1.4. Data Statistics and Analysis

The same experiment 1.

### 3.2. Research Findings and Analysis

#### 3.2.1. Baseline Test Comparison Analysis Between Groups Before Intervention

An independent samples *t*-test was performed on the pretest data of rhythmic perception abilities across sensory channels in both the experimental and control groups. The results showed no significant differences between the two groups in the duration of somatosensory modality rhythmic perception tasks, accuracy and reaction time of visual channel rhythmic perception tasks, or accuracy and reaction time of auditory channel rhythmic perception tasks (Table 3), indicating consistent baseline levels.

#### 3.2.2. The Effect of Dancesport on the Rhythm Perception Ability of the Body Sensory Channel in College Students

Repeated-measures ANOVA was used to investigate the effect of dancesport on the rhythm perception of college students’ somatosensory modality. The results showed that the main effect of group was not significant [*F*(1,38) = 0.35, *p* = 0.557, *η*^2^*_p_* = 0.01], the main effect of time was not significant [*F*(1,38) = 3.01, *p* = 0.091, *η*^2^*_p_* = 0.07], and the interaction effect of group and time was significant [*F*(1,38) = 10.40, *p* < 0.01, *η*^2^*_p_* = 0.22].

Simple effect analysis with Bonferroni-adjusted comparisons revealed that the post-test duration in the experimental group was significantly reduced compared to the pre-test (*p* = 0.001, adjusted, Cohen’s *d* = 0.43); no significant difference was observed between the control group in the pre-test and post-test (*p* = 0.70, adjusted, *d* = −0.13). In the pre-test, the duration difference between the experimental group and the control group was not significant (*p* = 0.84, adjusted, *d* = 0.07); similarly, the post-test difference remained statistically insignificant (*p* = 0.13, adjusted, *d* = −0.50).

**Figure 7:**
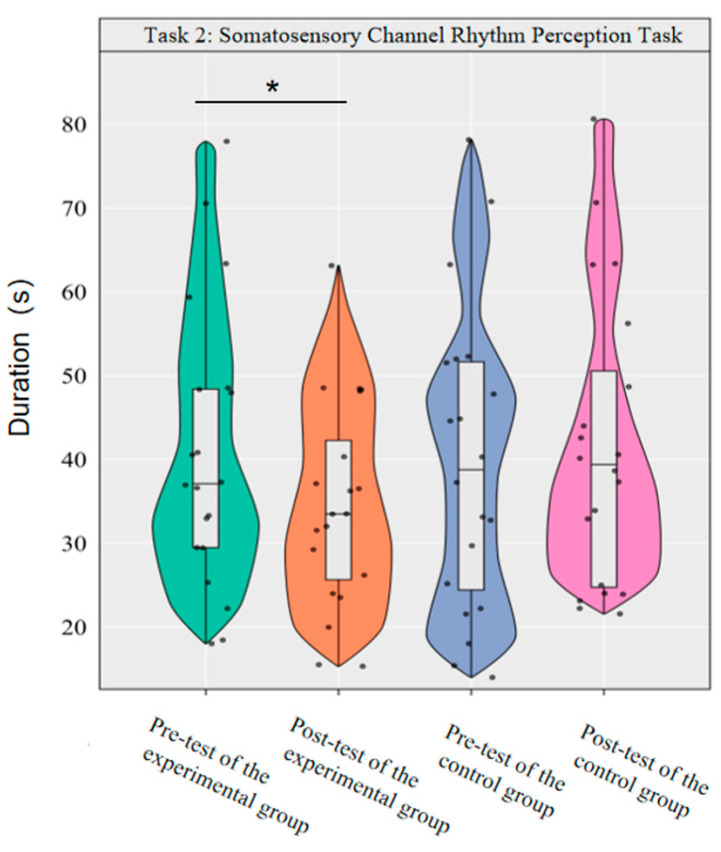
Comparison of the duration used by the two groups in the somatosensory modality before and after the test. Note: * *p* < 0.0125.

This demonstrates that a 10-week dancesport intervention can effectively enhance college students’ rhythmic perception in the somatosensory pathway, as shown by significant within-group improvement (Figure 7).

#### 3.2.3. The Effect of Dancesport on the Rhythm Perception Ability of College Students’ Visual Channel

A repeated-measurement ANOVA was conducted to investigate the effects of dancesport on the accuracy rate and reaction time of visual channel rhythm perception in college students. The results revealed significant main effects of group [*F*(1,38) = 4.49, *p* < 0.05, *η*^2^*_p_* = 0.11] and time [*F*(1,38) = 12.01, *p* < 0.01, *η*^2^*_p_* = 0.24] on the accuracy rate, as well as a significant interaction effect between group and time [*F*(1,38) = 4.42, *p* < 0.05, *η*^2^*_p_* = 0.10].

Simple effect analysis with Bonferroni-adjusted comparisons revealed that the post-test accuracy of the experimental group was significantly higher than that of the pre-test (*p* < 0.001, adjusted, Cohen’s *d* = 1.26); there was no significant difference between the control group in the pre-test and post-test (*p* = 0.30, adjusted, *d* = 0.38); in the pre-test, the difference in accuracy between the experimental group and the control group was not significant (*p* = 0.90, adjusted, *d* = −0.04); in the post-test, the accuracy of the experimental group was significantly higher than that of the control group (*p* = 0.002, adjusted, *d* = 1.05).

**Figure 8:**
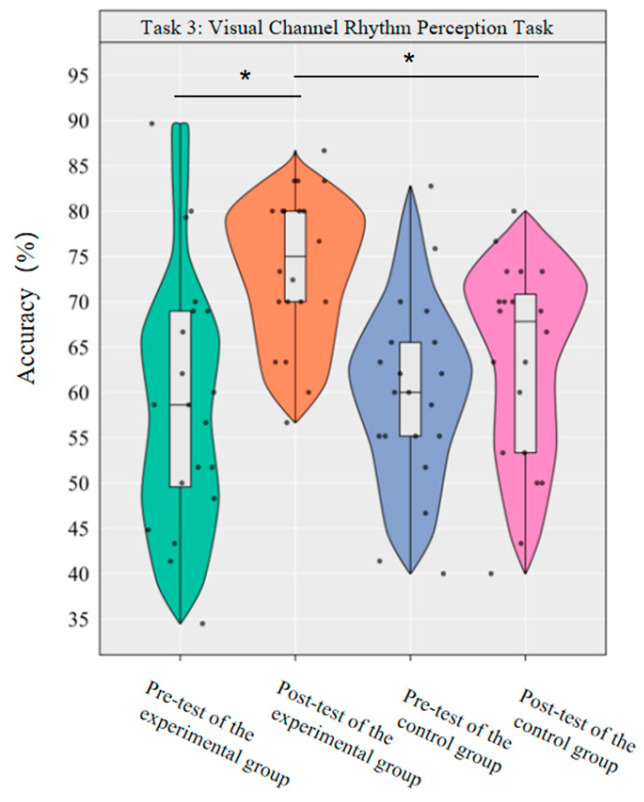
Comparison of the accuracy rates of the two groups in the visual channel before and after the test. Note: * *p* < 0.0125.

This demonstrates that a 10-week dancesport intervention can effectively improve the accuracy of visual channel rhythm perception among college students (Figure 8).

The main effect of group on reaction time was statistically significant [*F*(1,38) = 6.54, *p* < 0.05, *η*^2^*_p_* = 0.147], with the experimental group showing a significantly lower reaction time overall than the control group (Δ*M* = −336.37 ms). The main effect of time [*F* (1,38) = 0.70, *p* > 0.05] and the time × group interaction [*F* (1,38) = 1.01, *p* > 0.05] were not statistically significant. As the interaction was not significant, no further simple effect analysis was performed. Descriptive statistics revealed that the reaction time in the experimental group decreased by 207.49 ms post-test compared to pre-test (Cohen’s *d* = 0.44), while the control group showed a slight increase, though this difference was not statistically significant.

**Figure 9:**
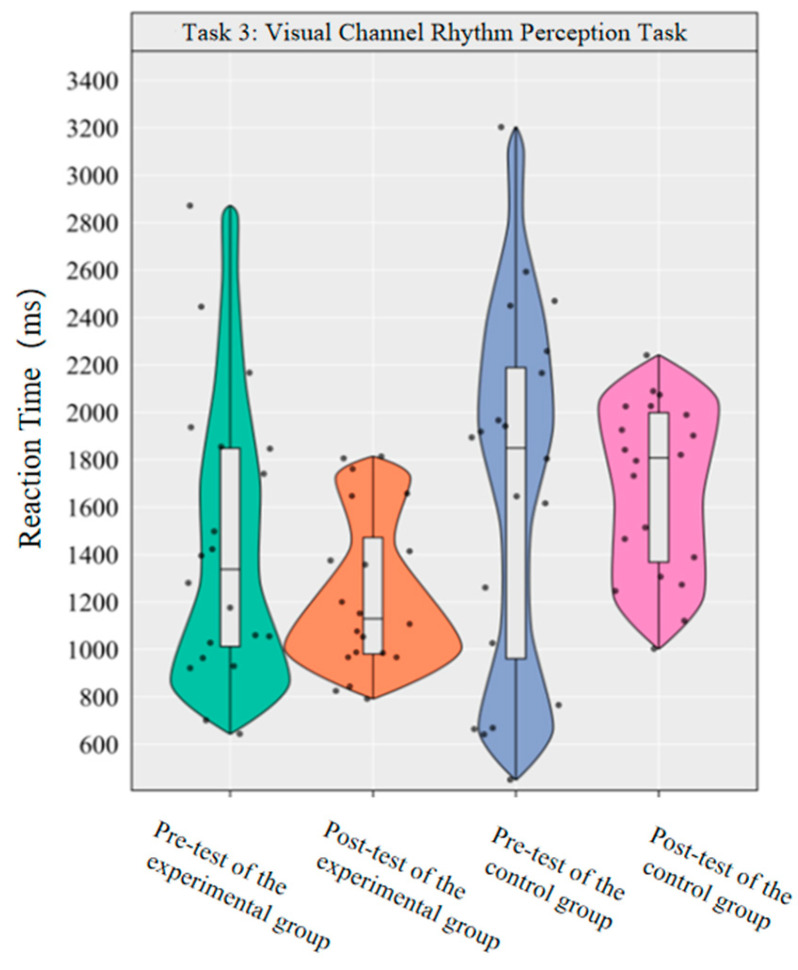
Comparison of reaction times before and after the experiment for the two groups of the visual channel.

This indicates that a 10-week dancesport intervention failed to effectively improve college students’ visual channel rhythm perception reaction time (Figure 9).

The results show that the 10-week dancesport intervention can effectively improve the accuracy of visual channel rhythm perception in college students, but has no significant effect on the reaction time of visual channel rhythm perception.

#### 3.2.4. The Effect of Dancesport on the Rhythm Perception Ability of College Students’ Auditory Channel

A repeated-measures ANOVA was conducted to investigate the effects of dancesport on the accuracy rate and reaction time of auditory channel rhythm perception in college students. The results revealed significant main effects of group [*F*(1,38) = 27.04, *p* < 0.001, *η*^2^*_p_* = 0.42] and time [*F*(1,38) = 8.76, *p* < 0.01, *η*^2^*_p_* = 0.19] on the accuracy rate, as well as a significant interaction effect between group and time [*F*(1,38) = 8.57, *p* < 0.01, *η*^2^*_p_* = 0.18].

Simple effect analysis with Bonferroni-adjusted comparisons revealed that the post-test accuracy of the experimental group was significantly higher than that of the pre-test (*p* < 0.001, adjusted, Cohen’s *d* = 1.30). There was no significant difference between the control group in the pre-test and post-test (*p* = 0.98, adjusted, *d* = 0.00); in the pre-test, the difference in accuracy between the experimental group and the control group was not significant (*p* = 0.31, adjusted, *d* = 0.29); in the post-test, the accuracy of the experimental group was significantly higher than that of the control group (*p* < 0.001, adjusted, *d* = 3.40).

**Figure 10:**
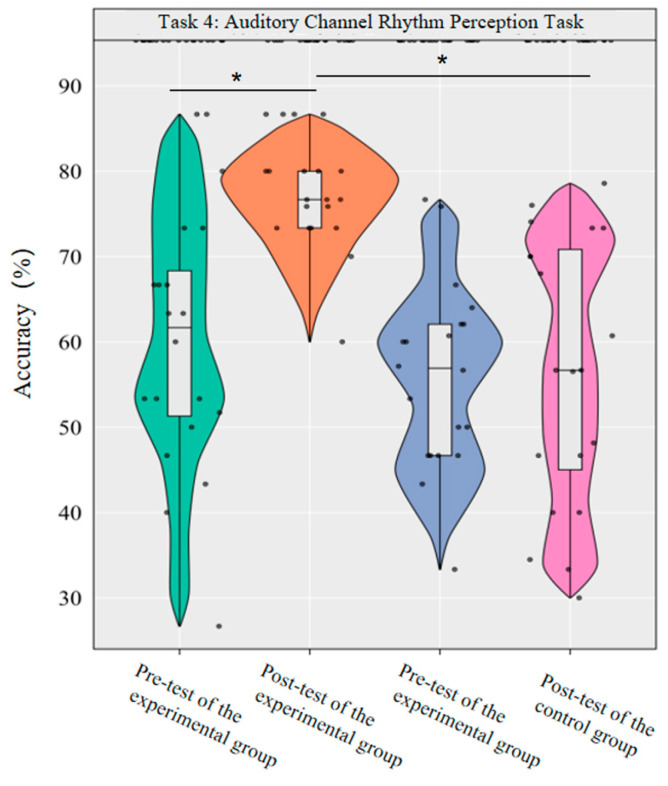
Comparison of the accuracy rates of the two groups in the auditory channel before and after measurement. Note: * *p* < 0.0125.

This demonstrates that a 10-week dancesport intervention can effectively improve the accuracy of auditory channel rhythm perception among college students (Figure 10).

In the reaction time index, the main effect of group was significant [*F*(1,38) = 26.17, *p* < 0.001, *η*^2^*_p_* = 0.41], the main effect of time was significant [*F*(1,38) = 24.74, *p* < 0.001, *η*^2^*_p_* = 0.39], and the interaction effect of group and time was significant [*F*(1,38) = 24.99, *p* < 0.001, *η*^2^*_p_* = 0.40].

Simple effect analysis with Bonferroni-adjusted comparisons revealed that the reaction time in the post-test of the experimental group was significantly reduced compared to the pre-test (*p* < 0.001, adjusted, Cohen’s *d* = 1.81); there was no significant difference between the pre-test and post-test in the control group (*p* = 0.99, adjusted, *d* = −0.33); in the pre-test, the difference in reaction time between the experimental group and the control group was not significant (*p* = 0.51, adjusted, *d* = −0.22); in the post-test, the reaction time of the experimental group was significantly lower than that of the control group (*p* < 0.001, adjusted, *d* = −1.76).

**Figure 11:**
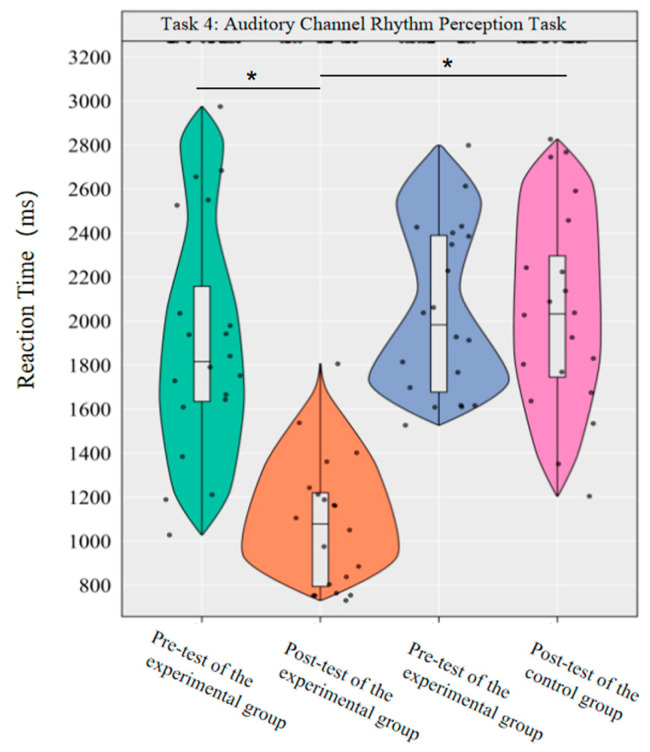
Comparison of reaction times before and after the experiment for the two groups of the auditory channel. Note: * *p* < 0.0125.

This demonstrates that a 10-week dancesport intervention can effectively improve college students’ rhythmic perception response time in the auditory pathway (Figure 11).

The results showed that the 10-week dancesport intervention could effectively improve the rhythm perception accuracy and reaction time of the auditory channel in college students.

## 4. Discussion

This study investigated two research questions: (1) Can dancesport training improve rhythm perception ability in college students? (2) Can dancesport enhance rhythm perception across multiple sensory channels in college students? Through a 10-week trial, we provide affirmative evidence for both. The first aim, to evaluate whether systematic dancesport training enhances rhythm perception capabilities, was achieved. The dancesport group demonstrated significant within-group improvements across all difficulty levels (pooled, medium, and high) with large effect sizes (Cohen’s *d* = 1.84, 2.08, and 1.33, all *d* > 0.8), and outperformed the active control group in the pooled-difficulty task post-intervention (Cohen’s *d* = 0.80, large). The second aim, exploring multisensory transfer, was also supported. Dancesport led to enhancements in somatosensory (Cohen’s *d* = 0.43, medium), auditory (accuracy *d* = 1.30, large; reaction time *d* = 1.81, large), and visual accuracy (*d* = 1.26, large), but not in visual reaction time.

### 4.1. The Effect of Dancesport on Rhythm Perception Ability of College Students

Our longitudinal intervention clearly demonstrates that compared to Health Qigong, a 10-week dancesport program significantly improves rhythm discrimination accuracy in college students. The effect was most robust for the pooled-difficulty score, with the experimental group surpassing the control at post-test with a large effect size (Cohen’s *d* = 0.80). Significant within-group gains with large effect sizes were also observed for both medium- (Cohen’s *d* = 2.08) and high-difficulty tasks (Cohen’s *d* = 1.33), though the between-group differences at these specific levels did not survive strict multiple comparison correction. This result was likely influenced by our modest sample size and increased variability in challenging tasks. The overall time by group interaction for the pooled-difficulty analysis also showed a large effect size (*η*^2^*_p_* = 0.19 > 0.14). This pattern suggests a reliable training effect that generalizes across task demands, potentially reflecting enhanced fine-grained temporal resolution.

This finding extends prior cross-sectional work showing rhythm perception advantages in expert dancers [15]. By providing causal, longitudinal evidence, we demonstrate that such advantages can be induced through training in novices. It aligns with and behaviorally corroborates intervention studies using other rhythm-focused activities [23]. The superior performance of the dancesport group over the Health Qigong control underscores the importance of obligatory auditory–motor synchronization. While both activities involved movement to music, only dancesport required precise and continuous alignment of complex motor sequences with an external rhythmic structure [24]. This demand likely engages and refines internal timing mechanisms and predictive processes more intensely, resulting in the observed large-magnitude behavioral gains.

We speculate that the core mechanism underlying this improvement may involve the continuous use of a predict and correct strategy. Dancesport, with its frequent rhythmic transitions and need for rapid movement adjustments, places participants under high temporal prediction pressure, potentially sharpening rhythmic acuity. Neurophysiological evidence supports this view. In an EEG study of ballroom dancers, Wang and colleagues found that dancers exhibited significantly stronger neural resonance to external rhythmic stimuli during audiovisual synchronization tasks compared to non-dancers, and that synchronization performance was modulated by beat interval [25]. This suggests that dance training can enhance the brain’s ability to entrain to and process fine temporal structures [26]. It is plausible that such enhanced neural resonance could contribute to the observed within-group improvement in our high-difficulty task, even though the between-group difference did not reach significance after strict correction. Notably, while Health Qigong is also a form of mind–body exercise performed with music, its rhythmic demands are qualitatively different from those of dancesport. Dancesport imposes a continuous requirement for precise predictive timing and error correction in synchrony with an external auditory rhythm [27]. In contrast, Health Qigong uses music primarily to establish a meditative context. Its movements follow an internal and self-paced flow rather than being locked to discrete musical beats. This critical distinction in the strictness of rhythmic coupling supports the interpretation that the observed improvements are specifically tied to the training of high-fidelity auditory–motor synchronization, rather than to general exercise or mindful movement alone.

This interpretation is consistent with research on sensorimotor synchronization, which indicates that the higher the coupling strength between action and rhythm, the more significant the improvement in rhythm perception ability [28]. Dynamical coupling theory further clarifies that when the coupling strength between the motor system (motor oscillation) and the auditory rhythm (perceptual oscillation) is higher (i.e., the better the match in phase and frequency), the brain’s ability to discriminate and synchronize with complex rhythms is enhanced [29]. The core of dancesport training is precisely to strengthen this coupling between motor and perceptual oscillations through high-intensity, high-precision guidance by external rhythms. This directly explains why the dancesport group achieved significantly better results than the control group in rhythm discrimination, particularly in the pooled-difficulty task (Cohen’s *d* = 0.80). This contrast supports a task-specific view of rhythm perception enhancement [30], where the degree of improvement is linked to the intensity and nature of the rhythmic synchronization required by the training.

### 4.2. The Influence of Dancesport on the Multi-Channel Rhythm Perception Ability of College Students

The results from Experiment 2 indicate that dancesport training can enhance rhythm processing across multiple sensory channels, with effects varying in magnitude and nature across modalities.

For the somatosensory modality, the dancesport group showed a significant reduction in the time needed to complete a rhythmic clapping and movement sequence from pre- to post-test (Cohen’s *d* = 0.43, medium). The time by group interaction for this measure was significant with a large effect size (*η*^2^*_p_* = 0.22 > 0.14). The improved performance on this task, measured as reduced time to complete the synchronized actions, indicates enhanced rhythm-driven action timing. This task required participants to continuously perceive the rhythm and translate it into precisely timed somatosensory–motor output. The finding suggests that dancesport training sharpened this perception-action translation loop specific to the somatosensory channel [25,31,32]. Unlike pure discrimination tasks, this paradigm captures rhythm perception as it is functionally used to organize movement, which is the core skill trained in dance. This within-group improvement aligns with the established role of the cerebellum and basal ganglia in motor timing and sensorimotor integration [29]. Our results extend previous research on musicians and dancers, which often shows superior sensorimotor synchronization [33,34], by demonstrating that such synchronization efficiency can be improved through structured training in novices. The fact that this improvement was primarily a within-group effect, without a strong between-group difference at post-test, may reflect the high variability inherent in this integrated perception-action measure or the relatively short training duration for inducing robust group-level differences in complex motor timing.

The most pronounced associations were observed in the auditory channel, where the dancesport group showed greater improvement in both accuracy (Cohen’s *d* = 1.30, large) and reaction time (Cohen’s *d* = 1.81, large) compared to the control group. The time by group interactions for both auditory accuracy and reaction time was significant with large effect sizes (*η*^2^*_p_* = 0.18 and 0.40, both > 0.14). This comprehensive enhancement highlights the unique role of integrated music and movement in dancesport. It corroborates and extends findings from studies on musical training, which consistently show advantages in auditory temporal processing [35]. More specifically, our results echo neuroimaging studies, which show that dance training can enhance the functional connection between auditory cortex and motor cortex [36]. We speculate that the continuous requirement in dancesport to align movements with musical beats may reinforce auditory–motor coupling [37], potentially contributing to the faster and more accurate perceptual decisions in pure auditory rhythm tasks observed here. This finding is consistent with theories of embodied cognition [38], which posit that cognitive functions like rhythm perception are shaped by sensorimotor experience. Research on multisensory rhythm perception further indicates that differences in sensory channel engagement across motor activities influence the extent of rhythm perception enhancement [7]. The superior promoting effect of dancesport on the auditory channel observed in our study corroborates this view, highlighting the efficacy of a training model characterized by high-intensity auditory–motor coupling in shaping rhythm processing advantages within specific sensory channels.

Regarding the visual channel, dancesport training improved discrimination accuracy (Cohen’s *d* = 1.26, large; time × group interaction *η*^2^*_p_* = 0.10, medium) but not reaction time. This dissociation suggests a dual-path mechanism for visual rhythm processing and invites comparison with existing literature. The significant improvement in visual accuracy aligns with findings that dancers show advantages in processing biological motion and rhythmic visual sequences [39]. It supports the view that dance training, which heavily relies on observing and imitating complex movement patterns, may enhance higher-order visual cognitive functions such as pattern recognition and working memory for temporal sequences. This could be mediated by increased functional connectivity in brain regions involved in action observation (e.g., the extended mirror neuron system) as suggested by research on dancers [12,40]. However, the lack of improvement in visual reaction time presents an interesting contrast. It suggests that while dance training optimizes the accuracy of interpreting visual rhythms, it may have a limited effect on the initial speed of visual processing or attentional capture by rhythmic stimuli. This distinction echoes findings in other domains where training improves accuracy but not necessarily speed of perceptual judgments [41]. It implies that the benefits of dancesport for visual rhythm processing are more closely tied to cognitive resources involved in decoding and memorizing temporal patterns than to low-level visual or attentional speed.

### 4.3. Research Perspectives and Limitations

While this study provides robust behavioral evidence, several limitations must be acknowledged, and they guide future research directions.

First and foremost, our interpretations regarding neural mechanisms (e.g., “neural circuit reshaping,” “auditory–motor network reorganization”) remain speculative, as we did not collect neuroimaging or physiological data. While our behavioral results are consistent with such mechanisms proposed in the literature [11,25], they do not provide direct evidence for them. Future studies should incorporate methods like functional magnetic resonance imaging, electroencephalography, or transcranial magnetic stimulation to test these hypotheses and elucidate the neural correlates of the observed behavioral changes.

Second, although our sample size was determined by an a priori power analysis and proved sufficient to detect the primary intervention effects, it remains modest. The attenuation of between-group effects under the most challenging conditions after multiple comparison correction suggests that a larger sample would provide greater statistical power to detect more subtle effects and enhance generalizability.

Third, the somatosensory rhythm task integrated perception with motor production. While this design captures the action-oriented nature of rhythm in this modality, it introduces a potential confound between perceptual improvement and gains in motor coordination or speed. Moreover, the reliance on a manual stopwatch for timing in this task constitutes a methodological weakness in terms of measurement precision. Manual timing is susceptible to human reaction error and lacks the millisecond accuracy achievable with computerized systems, which may have introduced uncontrolled variability and reduced the sensitivity of the measurement. Future studies should employ automated timing methods—such as motion capture, pressure-sensitive platforms, or software-based event recording—to obtain more precise, objective, and replicable assessments of sensorimotor synchronization efficiency. Future studies could include a control condition, for example, performing the same movements without rhythmic guidance, to better isolate the rhythm-specific perceptual component.

Fourth, the 10-week intervention period, while effective, is relatively short. Longer-term interventions and follow-up assessments are needed to determine the durability of these effects and whether continued training yields further gains.

Fifth, while we ensured no significant baseline differences in prior music, dance, or rhythm-game experience, we did not statistically control for these variables as covariates in our primary analyses. Future research could employ such statistical controls to obtain more precise estimates of the training effect, independent of pre-existing individual predispositions.

Sixth, as a holistic activity, dancesport concurrently trains motor skills, attention, and rhythm processing. While our use of a well-matched active control group (Health Qigong) strengthens the inference that gains are linked to the specific demands of rhythmic synchronization, the integrated nature of the intervention makes it challenging to isolate the unique contribution of each component. Future component-analytic studies are warranted to disentangle the specific effects of, for example, auditory–motor synchronization versus complex motor sequencing. Comparing a “rhythm-focused” training module with a “movement-focused” one could clarify the mechanisms behind the observed multisensory improvements.

In conclusion, this study demonstrates that a 10-week dancesport intervention can effectively enhance rhythm perception in college students across a range of difficulties and promote multisensory processing, with notable benefits in the auditory and somatosensory domains. These findings provide a scientific basis for considering dancesport as a viable multimodal cognitive training tool. Future work should focus on replicating these effects with larger samples, investigating the underlying neural mechanisms, exploring applications in clinical or educational populations, and designing optimized training protocols that target specific sensory channels.

## 5. Conclusions

This study designed four tasks to investigate whether dancesport can enhance college students’ rhythm perception abilities and simultaneously affect multiple sensory channels. The results demonstrated that a 10-week dancesport intervention effectively improved rhythm perception within the training group, as evidenced by significant within-group gains across pooled-, medium-, and high-difficulty levels. Critically, following the intervention, the experimental group also outperformed the control group in the post-tests of the pooled-difficulty rhythm perception tasks. Significant enhancements were also observed in both somatosensory and auditory channels compared to the control group. The improvement in visual channel rhythm perception was primarily reflected in accuracy rates, while reaction time showed no significant improvement.

## Figures and Tables

**Table 1:** Basic Information of the Subjects.

Class	Experimental Group (20 Individuals)	Control Group (20 Individuals)	*t*	*p*
Gender (Male:Female)	12:8	12:8	-	-
age (M ± SD)	20.60 ± 2.16	20.55 ± 1.73	0.081	0.936
Discipline of the major	3 STEM students Art category: 5 people 12 liberal arts students	9 STEM students Art category: 3 people 8 liberal arts students	−0.395	0.695
Do you have any sports hobbies?	12 with; 8 without	11 vs. 9	−0.789	0.435
Duration of sports activity (years) (M ± SD)	4.08 ± 2.64	4.67 ± 3.08	−0.466	0.646
Average weekly exercise duration (min/week) (M ± SD)	123.33 ± 56.62	175.50 ± 102.48	−1.512	0.146
MET-min/week	1276.80 ± 298.83	1554.30 ± 448.12	−0.515	0.221
Have you studied music-related specialties?	Yes: 7; No: 13	Yes: 6; No: 14	−0.330	0.744
Duration of music-related special skills training (years) (M ± SD)	3.43 ± 1.99	1.33 ± 0.58	1.739	0.120
Average weekly time spent learning music-related skills (min/week) (M ± SD)	118.57 ± 66.94	130.00 ± 121.24	−0.197	0.848
Have you studied dance-related skills?	Yes: 6; No: 14	4 yes; 16 no	−0.717	0.478
Duration of dance-related specialty training (years) (M ± SD)	5 ± 3.35	3.33 ± 1.16	0.814	0.442
Average weekly time spent learning dance-related skills (min/week) (M ± SD)	215.00 ± 209.93	210.00 ± 127.28	0.031	0.976
Do you have a habit of playing rhythm-based mobile games?	Yes: 4; No:16	5 people yes; 15 people no	0.370	0.714
Years of playing rhythm-based mobile games (M ± SD)	3.75 ± 4.19	5 ± 3.83	−0.440	0.675
Average weekly playtime for rhythm-based mobile games (min/week) (M ± SD)	82.50 ± 71.36	36.00 ± 32.86	1.310	0.231

**Table 2:** Test Results Before Rhythm Perception Intervention.

	Experimental Group	Control Group	*t*	*p*
Accuracy rate of difficulty and rhythm perception (%), not differentiated	47 ± 9	51 ± 6	−1.529	0.135
Correct rate of moderate difficulty rhythm perception (%)	40 ± 12	48 ± 13	−1.877	0.068
Accuracy rate of high-difficulty rhythm perception (%)	48 ± 13	54 ± 9	−1.887	0.067

**Table 3:** Differences in the pre-test of rhythm perception ability of each sensory channel between the two groups.

	Experimental Group (M ± SD)	Control Group (M ± SD)	*t*	*p*
Duration (s) for rhythm perception in the somatosensory modality	40.86 ± 16.72	39.72 ± 18.25	0.206	0.838
Visual pathway rhythm perception response time (ms)	1447.00 ± 596.51	1670.19 ± 770.93	−1.024	0.312
Visual pathway rhythm perception accuracy (%)	59 ± 14	60 ± 11	−0.125	0.901
Response time (ms) for rhythmic perception in the auditory pathway	1906.20 ± 826.45	2041.27 ± 387.32	−0.662	0.512
Accuracy rate of rhythm perception in auditory channels (%)	60 ± 16	56 ± 11	1.020	0.314

## Data Availability

The data presented in this study are available on request from the corresponding author. The data are not publicly available due to privacy and ethical restrictions.
